# Closed Flexor Tendon Avulsions Associated With a Distal Radius Fracture: A Case Report

**DOI:** 10.7759/cureus.96649

**Published:** 2025-11-12

**Authors:** Ross McAllister, Natan Silver, Daniel Brown

**Affiliations:** 1 Trauma and Orthopaedics, Warrington Hospital, Warrington, GBR; 2 Trauma and Orthopaedics, Aintree University Hospital, Liverpool, GBR

**Keywords:** avulsion, distal radius, flexor, fracture, tendon

## Abstract

Flexor tendon injuries have been reported in association with distal radius fractures. These are usually chronic in nature, and acute flexor tendon injuries are rare. When these do occur, they tend to be at the level of the fracture and are thought to be caused by direct laceration from the sharp bone edges of the fracture site. There are no reports of injuries more proximal to the fracture site at the musculotendinous junction. We present a patient who sustained a distal radius fracture with associated complete avulsions of the flexor digitorum superficialis tendons to the middle and ring fingers at the musculotendinous junction. This case demonstrates an unusual mechanism of injury to the flexor tendons and the importance of a comprehensive clinical assessment in high-energy distal radius fractures to ensure that this injury is recognised even when the flexor tendons at the level of the fracture appear to be in continuity.

## Introduction

Distal radius fractures are extremely common injuries; however, associated flexor tendon injuries are rare. When these do occur, they are most commonly chronic and attritional in nature, occurring months or years after the fracture [1]. Acute digital flexor tendon injuries accompanying distal radius fractures have been reported on very few occasions. They have all affected flexor digitorum profundus (FDP) tendons and have been attributed to lacerations on sharp fragments of bone [2].

Closed flexor tendon avulsions are a recognised pattern of injury in isolation. The majority occur at the tendon's distal insertion following forced hyperextension of a flexed digit [3]. Proximal avulsions at the musculotendinous junction are uncommon, making up just 5.1% of all flexor tendon injuries, and are thought to occur when the tendon's insertion is compressed, preventing failure, and so the force is transmitted further along to the musculotendinous junction [4]. We have found no reports in the literature of musculotendinous junction avulsions associated with distal radius fractures.

We describe a case of a high-energy, displaced distal radius fracture with associated avulsions of the flexor digitorum superficialis (FDS) to the index, middle, and ring fingers occurring at the musculotendinous junction, proximal to the fracture, along with a laceration of the index FDP at the level of the fracture.

We present this case to share our experience with this unusual injury and highlight the importance of a thorough clinical examination of patients with high-energy distal radius fractures to ensure that concomitant injuries are identified and treated appropriately, allowing optimal functional recovery.

## Case presentation

**Figure 1:**
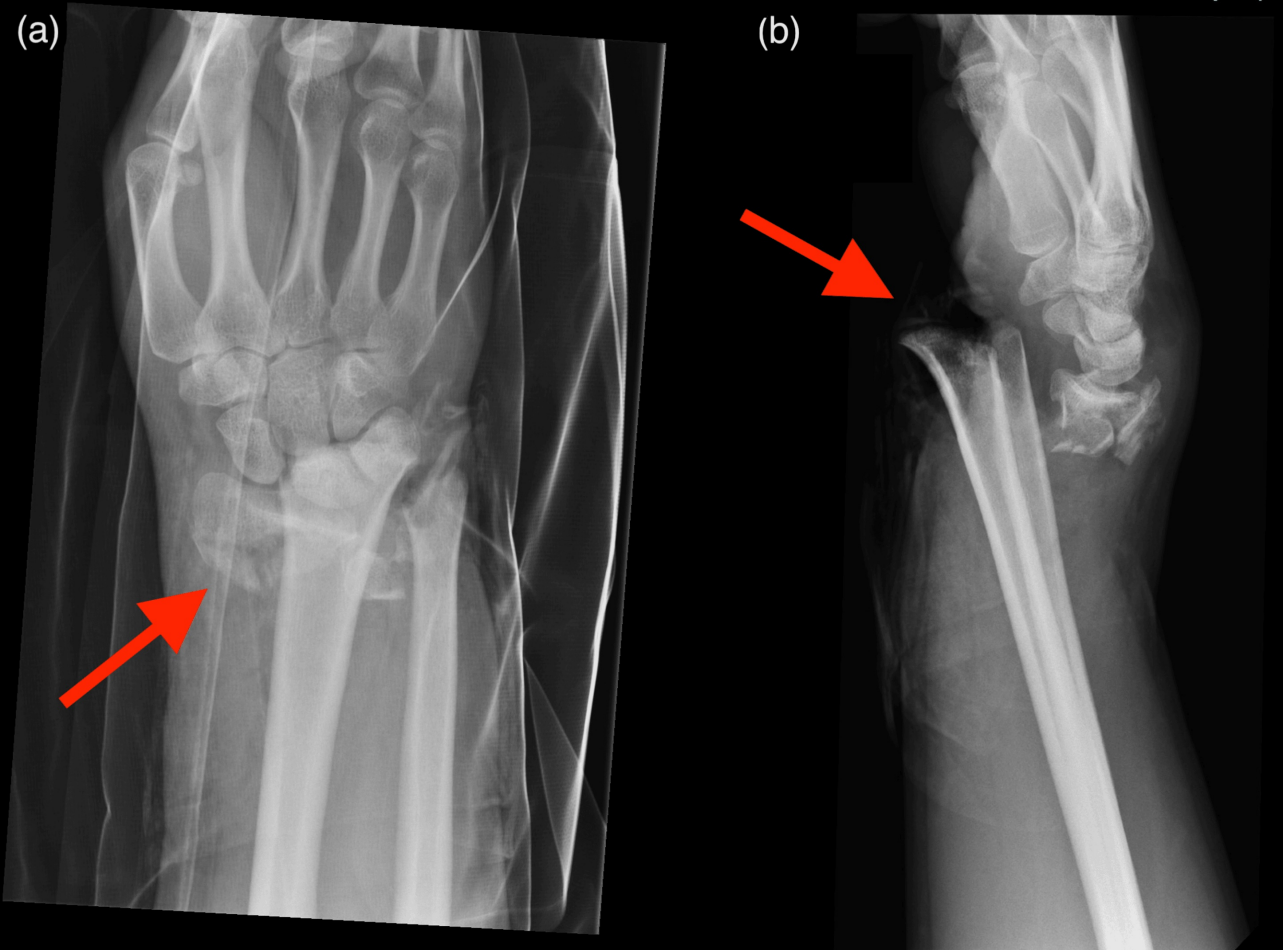
Initial radiographs PA (a) and lateral (b) radiographs demonstrating the severely displaced distal radius fracture (red arrows).

**Figure 2:**
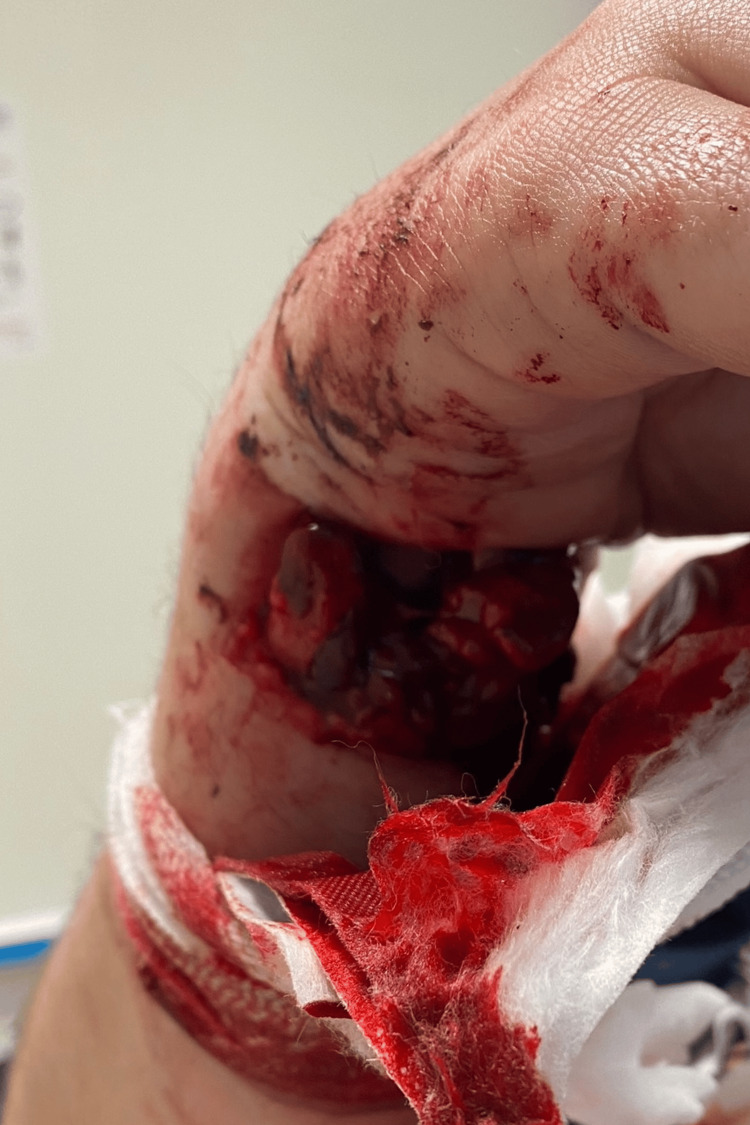
Large volar wound A clinical photograph of the wound on the volar aspect of the forearm at the level of the fracture with exposed bone.

A 30-year-old male was admitted to our institution following a high-energy motor vehicle accident. He sustained an open, severely displaced fracture to the right distal radius and ulna (Figure 1) with a Gustilo-Anderson type II 5 cm transverse volar wound just proximal to the wrist crease, at the level of the fracture, with the proximal fracture fragments visible in the wound (Figure 2). Examination revealed no neurovascular deficits, but there was a loss of the natural finger cascade and no active flexion at the PIPJ of the middle and ring fingers, suggesting a possible flexor tendon injury.

The patient was taken to the operating theatre, and a wound inspection revealed a partial laceration of the index FDP tendon at the level of the fracture. However, this did not explain the pre-operative clinical findings. Therefore, we extended the wound proximally and revealed complete avulsions of the FDS tendons to the middle and ring fingers at the musculotendinous junction and partial disruption of the FDS tendon to the index finger also at the musculotendinous junction, approximately 10 cm proximal to both the fracture and the laceration.

**Figure 3:**
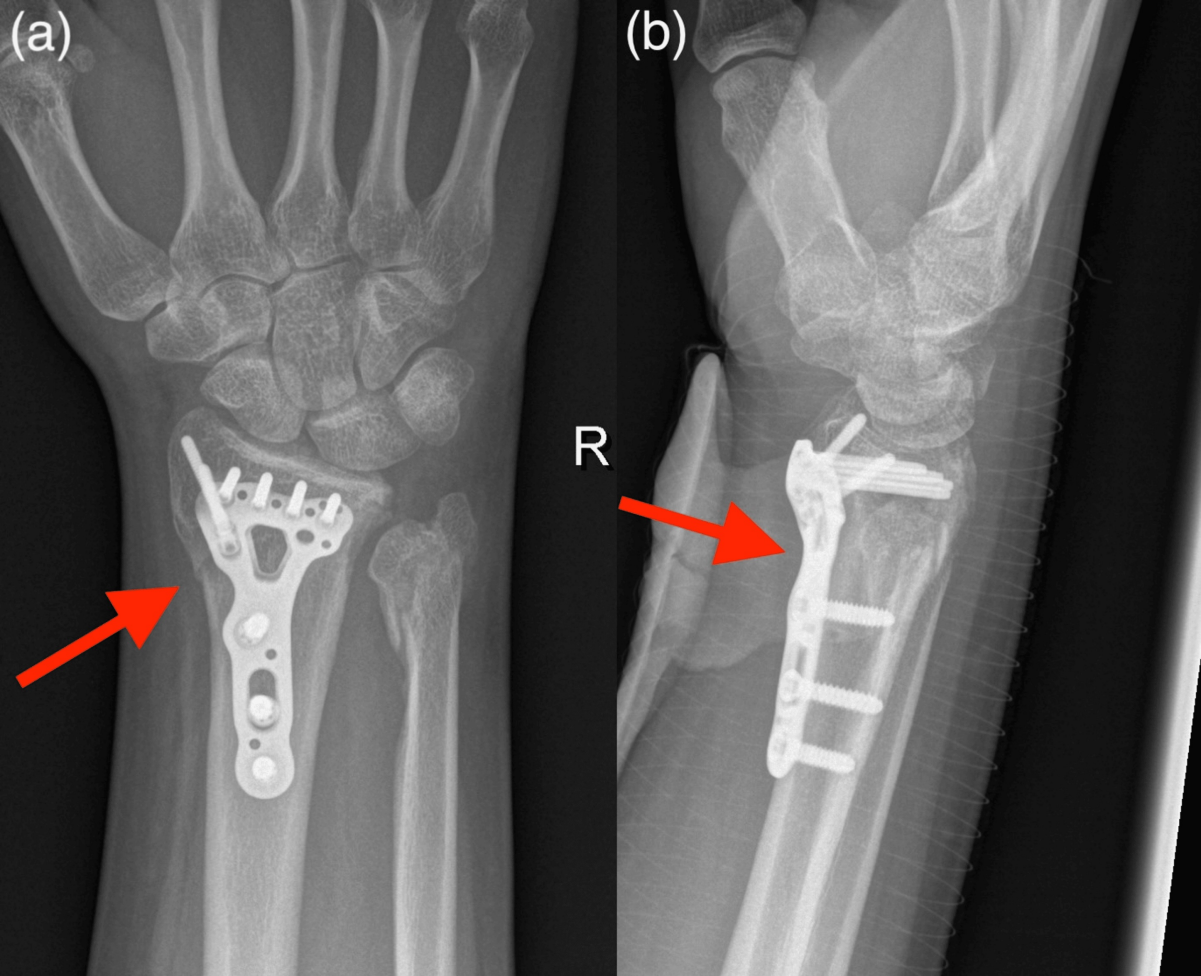
Postoperative radiographs PA (a) and lateral (b) radiographs demonstrating the surgical fixation (red arrows).

The fracture was anatomically reduced and fixed using an Acu-Loc 2 standard VDR plate (Acumed LLC, Hillsboro, OR) (Figure 3). The FDS avulsions of the middle and ring fingers were repaired onto the remnant tendon fibres within the muscle belly using the Adelaide technique and an epitendinous repair with an absorbable 3-0 Vicryl suture. The partial index FDP laceration was repaired in the same fashion. Intra-operative examination demonstrated restoration of the flexor cascade throughout passive wrist movement (the tenodesis effect). Therefore, the partial FDS avulsion to the index finger was not repaired.

**Figure 4:**
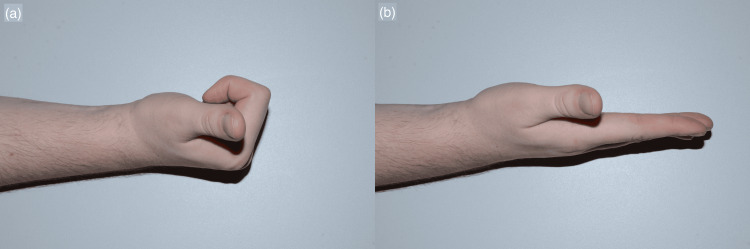
Functional outcome Clinical photographs demonstrating the functional recovery at 10 months post injury and the ability to achieve almost full composite flexion (a) and extension (b).

The patient underwent intensive hand therapy, following a controlled active motion protocol (early active motion with a resting splint in between). On follow-up at four months, he had achieved an almost full range of motion of all affected digits with normal hand function (Figure 4).

## Discussion

Although distal radius fractures are the most common fractures of the upper limb, associated tendon injuries are rare. This case demonstrates a previously undescribed pattern of tendon injury, characterised by avulsions of the FDS tendons at their musculotendinous junctions, in association with a distal radius fracture.

Closed flexor tendon ruptures have been classified initially by Vaughan-Jackson in 1969 [5] and later by Netscher in 2014 [6], who classified these based on their mechanism into traumatic tendon avulsions, spontaneous midsubstance rupture, infiltrative tenosynovial rupture, attrition rupture, and iatrogenic. Attrition ruptures can be further divided into non-traumatic and traumatic [7].

The majority of tendon complications following distal radius fractures are attrition ruptures after a malunion [8] or iatrogenic attrition ruptures on prominent metalwork [9], and they are usually chronic in nature. Acute flexor tendon injuries at the time of distal radial fractures are rare, with very few reported cases. There have been descriptions of acute injuries to the flexor carpi radialis, flexor pollicis longus, palmaris longus, and FDP tendons to the index and middle fingers [4,10]. To our knowledge, there have been no reported cases of an acute injury to the FDS tendons associated with a distal radius fracture.

It has been postulated that acute flexor tendon injuries associated with distal radius fractures are caused by direct laceration from the sharp fracture edge and are presumably uncommon due to the protective effect of the pronator quadratus lying between the bone and the flexor tendons [4,10]. This mechanism explains why the tendon injury occurs at the level of the fracture, as was seen in the partial FDP laceration to the index finger seen in this case. In contrast, the FDS avulsions in our patient occurred at the musculotendinous junction, well proximal to the fracture, and were presumably caused by a sudden tensile force acting across the musculotendinous junction during active muscle contraction, as the patient braced himself and gripped the steering wheel tightly, as his body was forced forward.

Acute spontaneous ruptures of healthy flexor tendons are exceedingly rare but have been reported, at the musculotendinous junction or in their midsubstance (in the palm or digital sheath), secondary to supramaximal strain or trauma, and are presumably the mechanism of injury seen in this case. They have never, to our knowledge, been previously reported in either the FDS tendon or in multiple tendons.

Repair of flexor tendon injuries at this level leads to satisfactory clinical results [11]. There is controversy as to whether repair of FDS tendons is necessary, with many surgeons of the opinion that the hand functions well as long as the FDP tendons are intact [12]. In this case, we elected to repair the complete FDS avulsions given the extent of the associated injury and the involvement of multiple tendons, in order to optimise function. The partial FDP injury was not repaired, as such injuries have been shown to achieve satisfactory function without repair [13], and the tenodesis effect was restored following repair of the complete injuries.

## Conclusions

This case highlights, to our knowledge, a previously unreported combination of a closed distal radius fracture with associated musculotendinous avulsions of the flexor tendons proximal to the level of the fracture. This pattern of injury could be easily missed, potentially leading to significant functional impairment. Appropriate clinical suspicion and a careful physical examination will ensure that acute tendon injuries are recognised and correctly managed, including evaluating the tendons at the musculotendinous junction if they are intact at the level of the fracture, leading to the best possible clinical results.
